# Propagation and Characterization of Influenza Virus Stocks That Lack High Levels of Defective Viral Genomes and Hemagglutinin Mutations

**DOI:** 10.3389/fmicb.2016.00326

**Published:** 2016-03-23

**Authors:** Jia Xue, Benjamin S. Chambers, Scott E. Hensley, Carolina B. López

**Affiliations:** ^1^Department of Pathobiology, School of Veterinary Medicine, University of PennsylvaniaPhiladelphia, PA, USA; ^2^Key Laboratory of Zoonosis of Ministry of Agriculture, College of Veterinary Medicine, China Agricultural UniversityBeijing, China; ^3^Wistar Institute and Department of Microbiology, Perelman School of Medicine, University of PennsylvaniaPhiladelphia, PA, USA

**Keywords:** influenza, defective viral genomes, defective interfering particles, hemagglutinin mutations, virus propagation and characterization

## Abstract

Influenza virus infections are responsible for more than 250,000 deaths annually. Influenza virus isolation, propagation, and characterization protocols are critical for completing reproducible basic research studies and for generating vaccine seed stocks. Detailed protocols for the isolation and identification of influenza virus have been recently reported (Eisfeld et al., 2014). However, there are few standardized protocols focused on the propagation and characterization of viral isolates, and as a result, viruses propagated in different conditions in different laboratories often have distinct *in vitro* and *in vivo* characteristics. Here, we focus on influenza A virus propagation and characterization in the laboratory taking into consideration the overall quality and composition of the virus stock to achieve consistency in virus yield, virulence, and immunostimulatory activity.

## Introduction

### Background

Influenza virus is a respiratory pathogen that can cause severe disease and even death in some individuals. According to the Centers for Disease Control and Prevention, influenza virus infects about 5–20% of the US population every year with more than 200,000 patients hospitalized. Different strains of influenza virus circulate among the population and variations of these strains arise regularly due to the high rate of mutations introduced by the viral polymerase (Salter et al., 2011; Eshaghi et al., 2014). In addition, new strains are sporadically generated due to re-assortment of the influenza virus genomic segments (Nelson et al., 2008; Danzy et al., 2014). This variability allows the virus to escape from the host immune response, permits multiple infections during the host lifespan, and promotes the generation of novel viruses that spread rapidly through the population.

Because of the significant impact of influenza virus in public health, multiple research groups around the globe are invested in researching influenza virus biology and pathogenesis. In addition, the influenza vaccine is adjusted annually to match the circulating virus strains and better protect the population. These efforts require frequent preparation of the virus in small (in the case of research laboratories) and large (in the case of vaccine manufacturers) scales. Lack of consistency in the methods utilized for influenza virus propagation and their characterization results in discrepancies in the yield, pathogenesis, and immunostimulatory properties of presumably identical strains of the virus (Marriott and Dimmock, 2010).

Two major influenza viruses circulate among the human population: influenza A virus (IAV) and influenza B virus (IBV). Since IAV is more broadly used in research laboratories, we will focus this article on IAV, however, similar principles and procedures apply to IBV. Based on our experience with IAV H1N1 and H3N2 subtypes, it is likely that this protocol applies to many influenza virus subtypes.

### Existing methods

IAV can be grown in 9–11 day old embryonated chicken eggs or in tissue culture in permissive cells, usually Madin-Darby Canine Kidney (MDCK) cells (Liu et al., 2009; Hussain et al., 2010) or kidney epithelium Vero cells, in the presence of trypsin (Tobita et al., 1975; Kaverin and Webster, 1995; Govorkova et al., 1999). Embryonated eggs or cells in culture are inoculated with a 10^−2^–10^−4^ dilution of a virus stock that could be originated from “seed virus” (i.e., a previous stock of virus propagated in the laboratory), a commercial source, or infected tissue (mouse lungs for mouse-adapted viruses or human respiratory secretions for human viruses). The virus is grown at 37°C for 40–72 h (sometimes longer) prior to collection of allantoic fluid or cell supernatants for storage and characterization. Embryonated chicken eggs have the advantage of producing high virus titers in a shorter period of time and these viral preparations are free of mammalian pathogens. Although there are safe well-characterized cell lines that are approved for generating influenza vaccines in some countries (Donis et al., 2014; Soema et al., 2015), low levels of contamination with mycoplasma or mammalian viruses are common in cell lines normally used to propagate influenza viruses for laboratory studies and their use is discouraged (David et al., 2010).

After growth, characterization of the virus stock is usually limited to determining the virus titer. Many laboratories estimate total viral particles in each preparation based on the amount of hemagglutinating units by calculating the end point dilution for hemagglutination of chicken, turkey, or guinea pig red blood cells (Killian, 2014). A caveat of this approach is that it does not distinguish infectious from non-infectious particles or fragments of viral or cell proteins containing the viral hemagglutinin protein. Titers of infectious units are usually calculated by counting viral plaque forming units (PFU) after infecting MDCK cells with different dilutions of virus, or by estimating the 50% tissue culture infectious dose (TCID_50_) (Szretter et al., 2006). Viruses prepared for vaccine purposes undergo additional more rigorous quality control processes to confirm the antigenic properties of the virus and, in particular, the antigenic quality of the surface HA and NA proteins (Wood and Robertson, 2004).

### The problem

**Figure 1:**
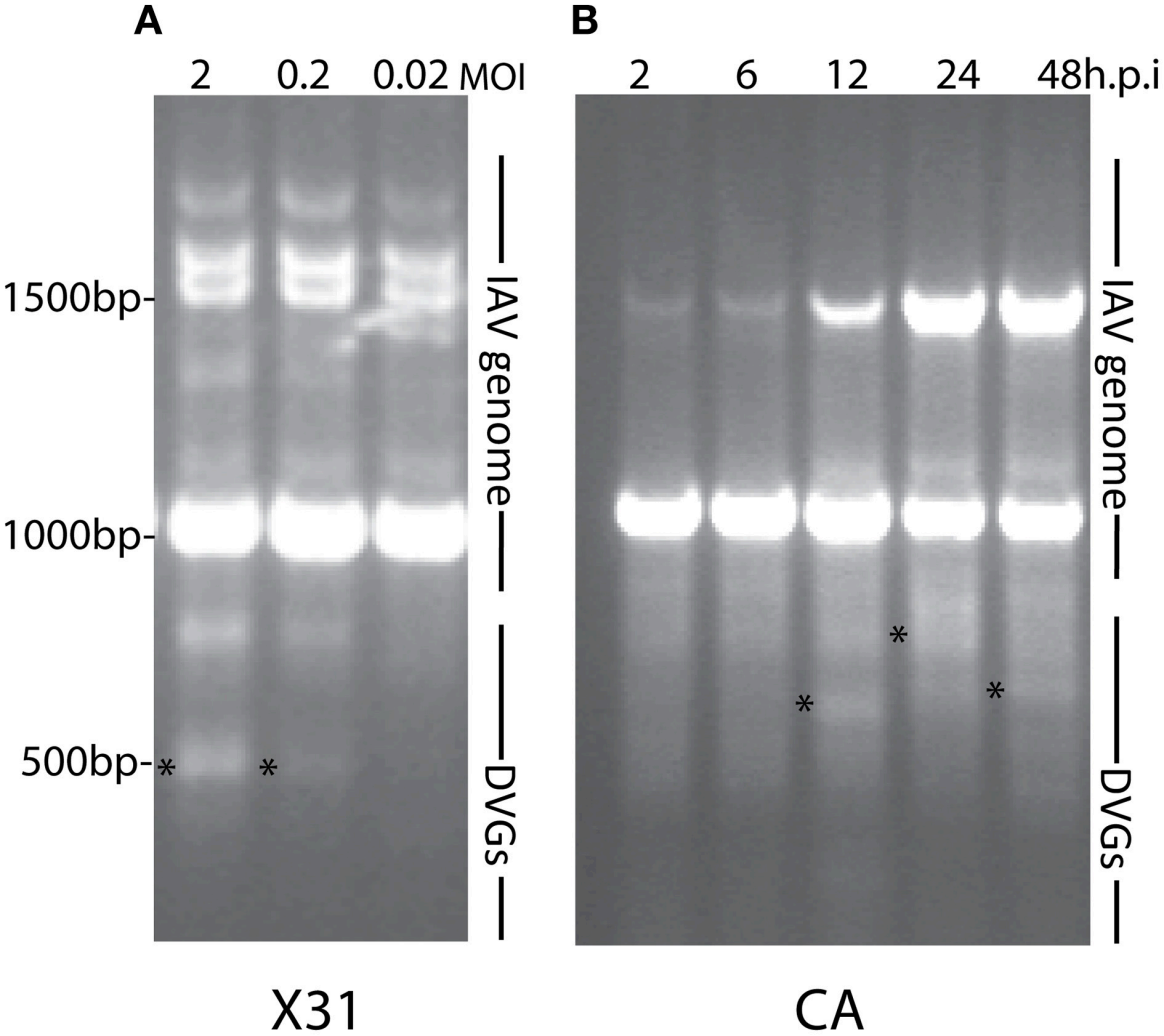
**DVGs accumulate in viral stocks when expanded at high multiplicity of infection (MOI) or extended periods of time**. As examples to illustrate the accumulation of DVGs in virus stocks propagated in different conditions **(A)** shows viral PCR products amplified from A549 cells infected with a MOI of 2, 0.2, or 0.02 of IAV HK/x31 (X31) (H3N2) for 6 h. **(B)** Shows viral PCR products amplified from A549 cells infected with a MOI 0.1 of A/California/7/2009 (CA) (H1N1) for 2, 6, 12, 24, or 48 h. Position of base pair size reference markers is indicated in each gel. Asterisks indicate DVGs confirmed by sequencing.

**Figure 2:**
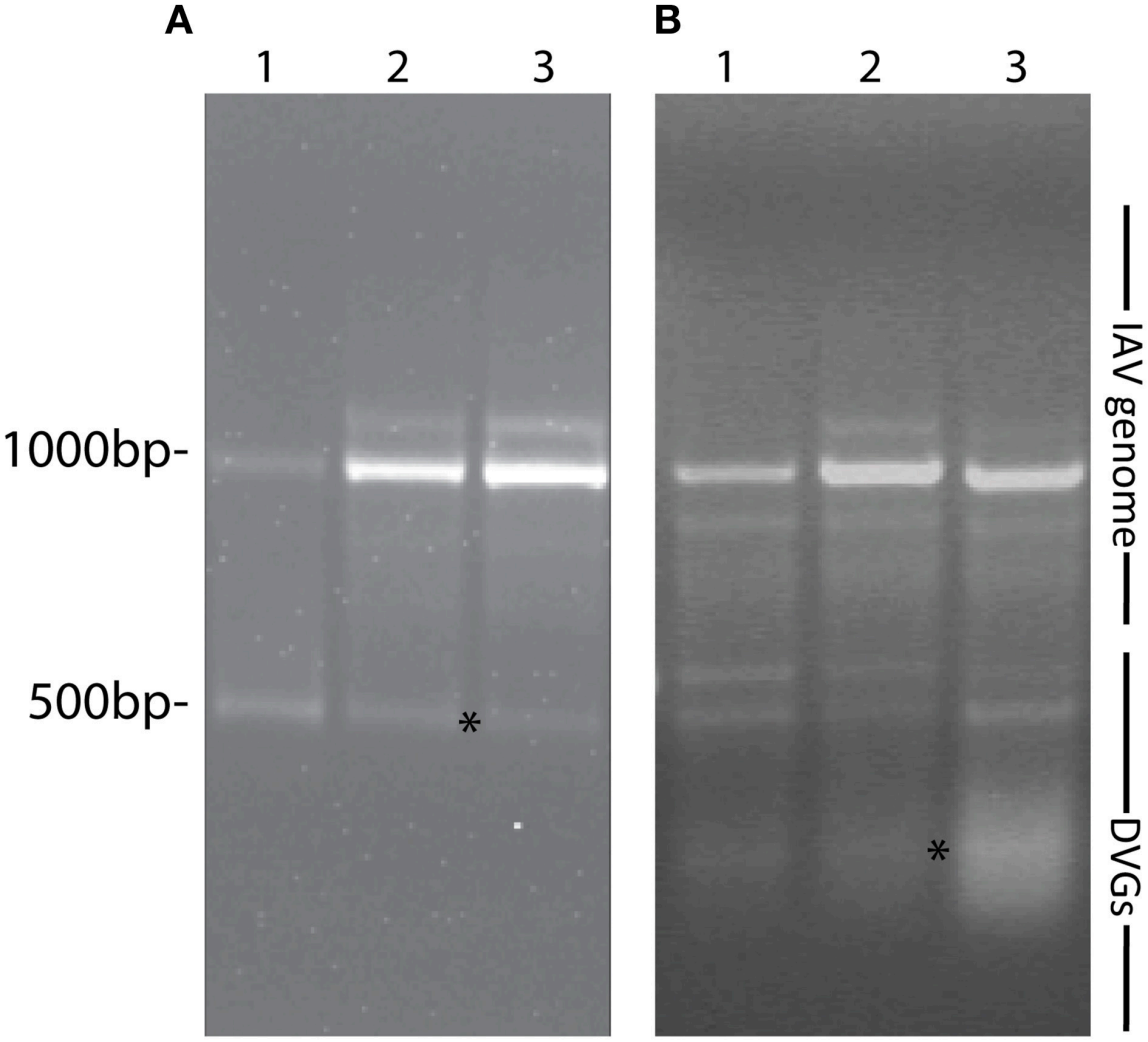
**IAV DVGs are found in live-attenuated influenza vaccines. (A)** PCR products detecting DVGs in live attenuated influenza vaccine stocks. Lanes 1, 2, and 3 represent live influenza vaccine NR-17600 intranasal spray formula for the 2009–2010 season, live influenza vaccine NR-21987 intranasal spray formula for the 2010-2011 season, and live influenza vaccine NR-36465 intranasal spray formula for the 2011-2012 season, respectively. **(B)** PCR products identifying DVGs in A549 cells infected with the different vaccine stocks. Vaccine isolates were obtained from BEI Bioresources. Position of base pair size reference markers is indicated in each gel. Asterisks indicate DVGs confirmed by sequencing.

Most research groups do not take in consideration variations in the composition of the viral isolates that are used to create influenza preparations and/or the modifications introduced to the virus during imprecise propagation conditions (Robertson et al., 1993; Marriott and Dimmock, 2010). Virus replication generates multiple products in addition to full-length standard viruses. A major byproduct is a set of severely truncated viral genomes collectively named defective viral genomes (DVGs) for their inability to replicate without a standard virus (Von Magnus, 1954; Lazzarini et al., 1981; Perrault, 1981; Lopez, 2014). IAV DVGs were first described by von Magnus in the late 1940s after growing viruses at high multiplicities of infection (MOI) (Von Magnus, 1951). IAV DVGs contain at least one deletion in one of the virus genomic segments (Hutchinson et al., 2010; Scott et al., 2011) and IAV DVGs with different activities have been described (Brooke, 2014). Although no consensus exists whether deletions in specific viral segments confer specific properties to the virus, IAV DVGs with the ability to interfere with the replication of the standard virus have been mostly observed as deletions in the genomic segments encoding the polymerase genes (PA, PB1, and PB2) (Noble and Dimmock, 1995). Deletions in the genomic segment that encodes for the NS1 protein, which strongly antagonizes the host antiviral response (Ngunjiri et al., 2013), leads to the production of viruses able to induce high levels of type I interferon (IFN) (Ngunjiri et al., 2012). Because of their interference activities, DVGs are also called defective interfering particles (DIPs). IAV DVGs have been found in viral stocks generated during growth in both embryonated eggs or in cell culture. DVGs are generated under a wide range of conditions that include high MOI and extended growth periods (Von Magnus, 1951; Magnus, 1954; Janda et al., 1979). Data obtained in our laboratory illustrating the impact of growing conditions in the accumulation of DVGs in virus stocks is shown in Figure 1. Remarkably, we also found that live-attenuated IAV vaccine stocks of recent years contain DVGs (Figure 2).

**Figure 3:**
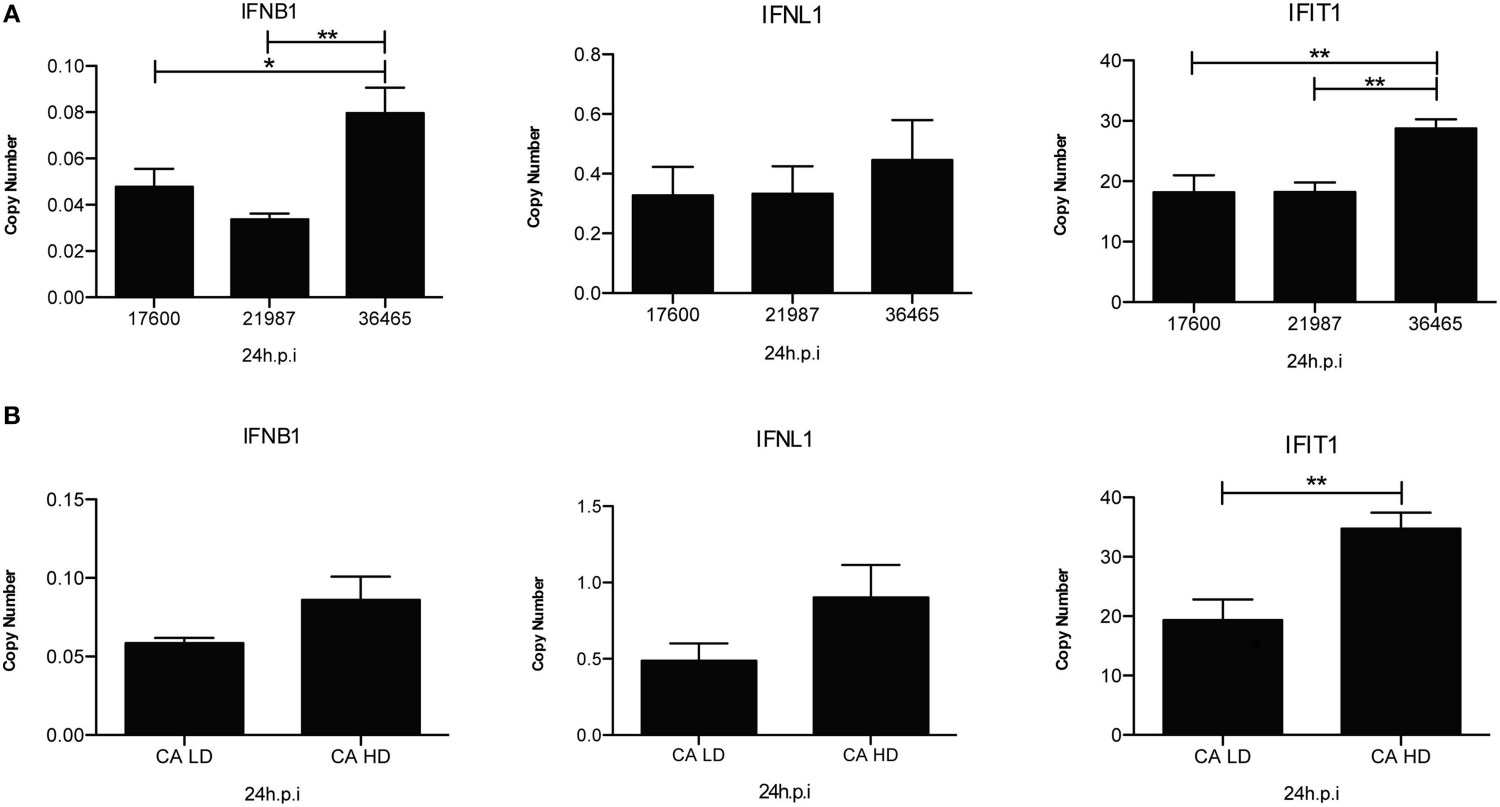
**IAV isolates with a high content of DVGs induce stronger antiviral responses in infected cells. (A)** A549 cells were infected with live, attenuated influenza vaccines at 2.5 fluorescent focus units (FFU) per cell for 24 h. Expression of *IFNB1, IFNL1* (IL29), and *IFIT1* (ISG56) was examined by RT-qPCR and is shown as copy number relative to the housekeeping genes β-actin and GAPDH. The numbers under each histogram bar correspond to one influenza vaccine isolate (NR-17600, NR-21987, or NR-36465) (^*^p < 0.05, ^**^p < 0.01 by one-way ANOVA post Bonferroni's multiple comparisons). **(B)** A/California/7/2009 viruses with low DVGs (LD) and high DVGs (HD) content were used to infected A549 cells (MOI = 0.5). Gene expression was analyzed after 24 h (^**^p < 0.01 by two-tailed *t*-test). All error bars indicate mean ± SEM of three independent experiments.

A large body of evidence demonstrates that infection of mice with IAV preparations with a high content of DVGs protects from pathology and diminishes viral load (Noble and Dimmock, 1995; Dimmock et al., 2008; Saira et al., 2013; Tapia et al., 2013; Boergeling et al., 2015). The protection conferred by IAV DVGs is largely mediated by type I IFNs demonstrating an enhanced immunostimulatory activity for virus stocks containing high levels of DVGs (Janda and Nayak, 1979; Frensing et al., 2014). Examples of the impact of DVGs in the antiviral response are shown in Figure 3.

Minor viral mutations generated during virus expansion include the acquisition of HA mutations that facilitate efficient replication in eggs and cell cultures. Many of these mutations affect the antigenic properties of the virus (Katz and Webster, 1989; Robertson et al., 1993; Rocha et al., 1993). In addition, a vaccine mismatch that occurred in the 2012–2013 influenza season resulted almost exclusively from an HA mutation that emerged when the H3N2 vaccine strain was propagated in chicken eggs (Skowronski et al., 2014).

Standardizing the procedures to prepare, propagate, and characterize virus stocks focusing on maintaining the quality of the virus in regards of their composition, yield, pathogenesis and immunostimulatory properties is essential for reproducible research and consistent preparation of vaccine stocks.

## Overview of the procedure

A detailed procedure for the isolation and identification of IAV was recently reported (Eisfeld et al., 2014). The protocol described here focuses on the standardization of the conditions for virus propagation in the laboratory and on characterization of the virus stocks. This protocol provides quality control procedures to characterize the DVG content of viral stocks and includes a strategy to prepare human IAV isolates that can grow in eggs with minimal HA mutations. These methods have been used in previous publications (Tapia et al., 2013; Linderman et al., 2014). Although this is not a clinical protocol, the procedures described are applicable for the propagation and characterization of viruses isolated from clinical samples, as well as for the preparation of virus stocks to be used in the annual preparation of the influenza vaccine. This protocol also provides the methods for DVG detection and identification. It is important to note that the principles discussed here also apply to the propagation of other RNA viruses, albeit specific details may vary.

### Virus isolation

According to current regulations influenza virus research should be conducted in laboratories with at least PC2/BSL2 standards and virus handled in a class II biological safety cabinet. Working with highly pathogenic viruses requires higher biosafety levels.

**Figure 4:**
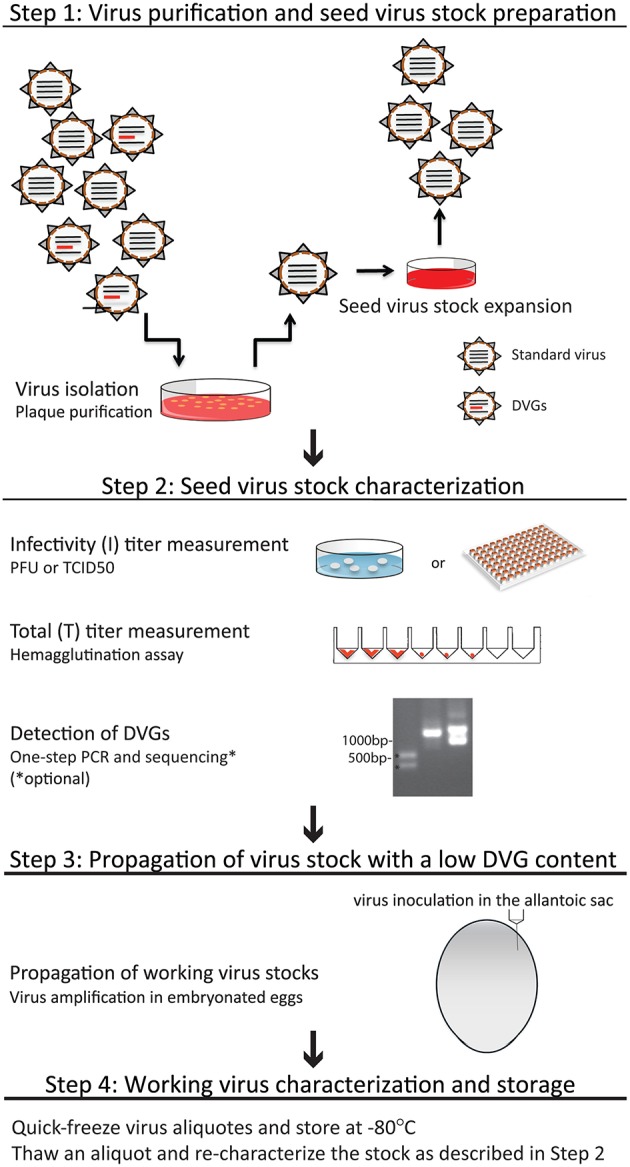
**Summary of the protocol for generation of viruses with a low DVG content**. Details of each procedure can be found in the text.

The steps in this protocol are summarized in Figure 4. Regardless of the virus source, seed virus should be initially grown from a single virus clone isolated by plaque purification. This approach minimizes contamination of the initial seed virus with DVGs. Plaques are the result of virus cytopathic effect in the monolayer and are generated from the expansion of a single virus particle and the spread of progeny virus to cells immediately next to the originally infected source. If the initial titer of the seed virus is unknown, it is recommended to perform serial dilutions of the virus to obtain isolated plaques. In brief, an 80 to 90% confluent monolayer of MDCK cells is seeded with a virus inoculum of 10–50 infectious particles per million cells diluted in the minimum volume of infection media (IM) necessary to fully cover the cell monolayer. This inoculum size/cell volume permits isolation of individual plaques. Cells should test negative for mycoplasma and other pathogens before use. After virus adsorption, IM is carefully aspirated from the cells. The infected cell monolayer should then be washed gently with PBS and overlaid with media containing agarose and TPCK-trypsin to promote multicycle replication. The agarose limits virus spreading in the plate. The overlay media should be maintained at 45–46°C as the cells will detach if the temperature is too high or the overlay media will be solidified before use if the temperature is too low. After adding the overlay media to the plates at room temperature, plates should be incubated at 37°C, 7% CO_2_ for 72 h (Note: our laboratory uses 7% CO_2_ in our tissue culture incubator, however 5% CO_2_ is also appropriate). Plaqued virus is then collected by pinching through the agarose layer with a 1 ml sterile micropipette tip. Each agarose plug is collected in a separate microcentrifuge tube containing 1 ml sterile and ice-cold 0.1% gelatin in PBS. If the plaqued virus will be expanded on the same day of collection, the virus can be stored at 4°C. Otherwise, the plaqued virus stock should be aliquoted and quick-frozen in a dry ice/ethanol bath and stored at −80°C.

### Preparation of a seed virus stock

Plaqued virus can be expanded to prepare a seed virus stock by inoculating a fraction of the preparation into plates seeded with MDCK cells or into embryonated chicken eggs (details below). Inoculated MDCK cells should be incubated for 24 h in the presence of TPCK-trypsin. The chicken eggs allantoic fluid should be harvested 40 h after injection. Longer incubation times will promote the generation of DVGs. Supernatants containing seed virus are collected and centrifuged to remove debris before aliquoting and quick-freezing for storage at −80°C.

### Seed virus characterization and quality control

Before proceeding to expand the seed virus into a working stock, the virus should be tested for DVGs. An initial estimation of the presence of defective genomes can be obtained by determining the ratio between infectious particles and total viral particles in the preparation. Infectivity titer (I) measures only complete virus particles able to replicate, while total titer (T) measures all virus particles even those with incomplete or truncated segments. As different strains of IAV virus accumulate DVGs at different rates, this procedure is only useful if the expected ratios are known. Table 1 shows examples of I/T ratios in different IAV strains with a low (LD) or high (HD) content of DVGs. Virus containing DVGs have 3–20 times lower ratios than viruses lacking DVGs.

**Table 1:** **I/T ratios in different IAV strains with a low (LD) or high (HD) content of DVGs**.

**Virus**	**Status**	**Infectivity titer (I): (log10)TCID_50_/25 μl**	**Total titer (T): HA/25 μl**	**I/T**
A/Puerto Rico/8/1934	LD	8.4	2048	120 × 10^4^
A/Puerto Rico/8/1934	HD	7.7	1024	4.9 × 10^4^
A/New Caledonia /03/2005	LD	6.4	256	10,000
A/New Caledonia /03/2005	HD	5.9	256	3000
A/California/7/2009	LD	4.7	64	800
A/California/7/2009	HD	4.4	128	200

### Infectivity (I) titer measurement

Two methods can be used to determine the amount of infectious viral particles in the stock: 50% tissue culture infectivity dose (TCID_50_) and plaque assay (plaque-forming units; PFU) (Szretter et al., 2006). In brief, to determine TCID_50_,1/10 dilutions of the virus stock are seeded in triplicates or quadruples using the Reed and Muench method (Reed and Muench, 1938). The virus is seeded over a monolayer of MDCK cells and incubated in the presence of TPCK-trypsin for 72 h at 37°C, 7% CO_2_ (Note: our laboratory uses 7% CO_2_ in our tissue culture incubator, however 5% CO_2_ is also appropriate). Cytopathic effect or the presence of virus in the supernatant (as determined by hemagglutination assay as described below) is used to determine end point dilutions. To determine PFU, virus dilutions are seeded over a monolayer of MDCK cells and incubated in the presence of overlay media containing agarose and TPCK-trypsin for 72 h at 37°C, 7% CO_2_. After removal of the overlay media (agarose), the monolayer is stained with crystal violet and plaques are counted.

### Total (T) titer measurement by the hemagglutination assay

Hemagglutination, the ability of the viral HA protein to bind and agglutinate red blood cells, depends on the amount of HA protein on the surface of membranes. The hemagglutination titer is calculated based on the end point dilution of virus able to agglutinate a set concentration of chicken, turkey, or guinea pig red blood cells (Szretter et al., 2006).

### Detection of DVGs by PCR

IAV DVGs contain at least one truncated genomic segment in the virus particle. A PCR assay that amplifies all eight influenza virus segments using a universal set of primers, or PCR assays using specific set of primers for each viral genomic segment, should be used to determine the presence of DVGs in the virus stock (Zhou et al., 2009; Frensing et al., 2014). Upon extraction, cloning, and sequencing of the amplicons, this method also allows the identification of the specific defective viral genome. The majority of the IAV DVGs described to date are around 300–700 bp long. Amplicons of that size should be cloned and sequenced to confirm the presence of DVGs.

### Propagation of virus stocks maintaining a low DVG content

DVG-free seed IAV is propagated in 9–11 day old embryonated chicken eggs to generate a working virus stock. Each egg produces an average of 8 ml of virus-containing allantoic fluid. It is essential to carefully follow established virus growth conditions to avoid the generation of DVGs during propagation. Egg inoculations with highly concentrated virus or extended growth periods should be avoided. Details of the process of egg inoculation were recently described (Szretter et al., 2006; Eisfeld et al., 2014). To maintain low levels of DVG, eggs are inoculated using aseptic techniques with no more than 30,000 TCID_50_ of the seed virus diluted in 100 μl of PBS. Inoculated eggs are incubated at 37°C with 45% humidity for 40 h. Allantoic fluid is then collected after euthanizing the embryo at 4°C for 3–4 h. Allantoic fluid should be clear; a reddish color indicates red blood cell contamination and a cloudy color indicates bacterial contamination. If the yolk sac is disrupted (yellowish color) the allantoic fluid should be discarded. Each egg should be collected in separate sterile tissue culture tubes and the allantoic fluid should be tested for bacterial contamination by inoculating trypticase soy agar (TSA) plates with sheep blood. Clean allantoic fluids harvested from different eggs are then pooled, mixed, aliquoted, and frozen at −80°C. Alternatively, IAV can be propagated in clean, mycoplasma-free MDCK or Vero cells by inoculating 80 to 90% confluent monolayers of cells with virus at MOI of 0.1 and incubating for 24 h at 37°C in a tissue culture incubator. Virus is collected in the cultures supernatants and centrifuged to remove debris and aliquoted and frozen at −80°C.

### Virus stock storage

Allantoic fluid or infected cells supernatant is snapped frozen in a dry ice/ethanol bath, aliquoted, and stored at −80°C. To maintain the virus infectivity titer, repeated freezing and thawing must be avoided. Once thawed, virus stocks are kept on ice or at 4°C and used the same day.

### Working virus stock characterization

Working virus stock should then be titrated and analyzed for DVG content as described above before use. As discussed, many influenza virus isolates will acquire adaptive mutations when propagated in eggs and cell culture. This can especially be a problem with primary human influenza virus isolates. Virus genomes should be routinely sequenced to determine if adaptive mutations arose during propagation. Although adaptive mutations can arise in any gene segment, mutations in HA are common egg adaptations. At the very least, the HA gene of each viral preparation should be sequenced. PCR followed by standard Sanger sequencing can detect mutations that are abundant in viral preparations, while Illumina sequencing is recommended to detect minor adaptive variants.

### Reverse-engineering viruses so that they can grow in eggs and cell culture

**Figure 5:**
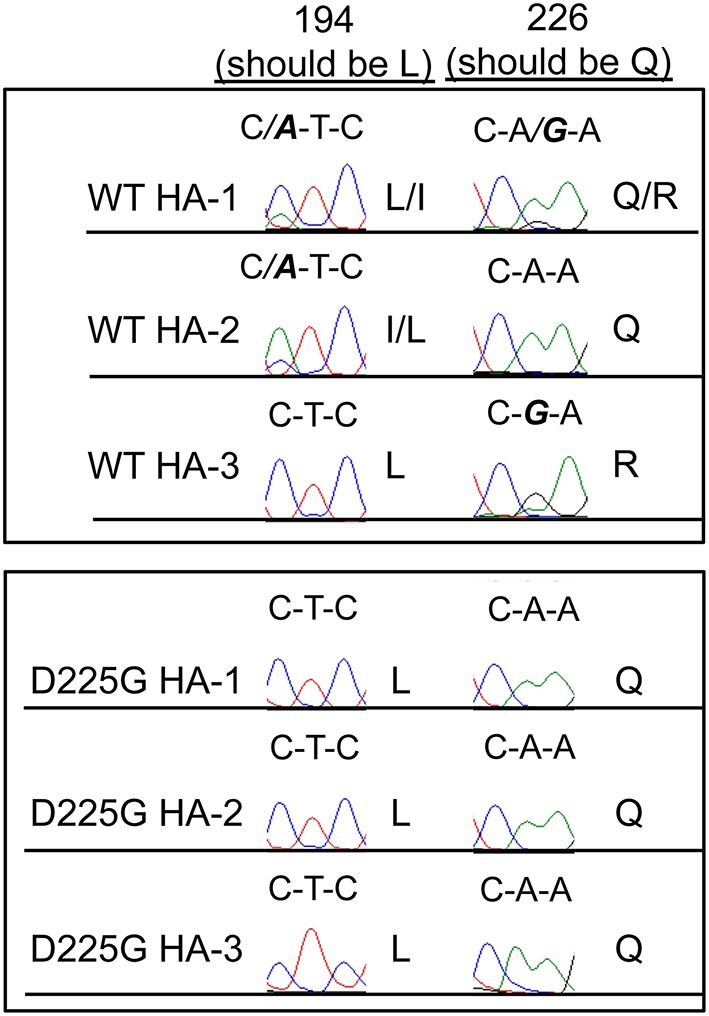
**Limiting HA mutations in engineered viruses**. Three different stocks of wild-type (WT) A/California/7/2009 H1N1 viruses were grown in eggs. Three stocks of A/California/7/2009 engineered to possess the D225G HA mutation were also grown in eggs. We sequenced the HA genes of these viral preparations by Sanger sequencing. Shown are sequencing results of nucleotides that encode HA residues 194 and 226. All WT preps possessed mutations, but mutations were undetectable throughout the entire HA gene isolated from viruses engineered to have the D225G HA mutation (other than the desired D225G HA mutation). Mutations are shown in bold and italics.

Some viral isolates cannot be grown in eggs or cell culture without acquiring adaptive HA mutations. When confronted with this situation, there are at least two options. The first option, used by many, is to simply passage the virus in eggs until high titer viral stocks are obtained. Using this approach, viruses acquire different adaptive mutations and there is often a mixture of mutant viruses in these preparations. The second option, that we routinely use, is to use reverse genetics to introduce specific mutations that promote viral growth in eggs or cell culture. A detailed description of the reverse-genetics process has been previously reported (Neumann et al., 2005; Martinez-Sobrido and Garcia-Sastre, 2010). Strategically introducing specific adaptive mutations allows the experimentalist to control the types of adaptive mutants that will be present in the viral preparation. For example, the A/California/7/2009 H1N1 strain is difficult to grow in eggs and cell culture because the HA of this virus does not bind well to sialic acid receptors on eggs or MDCK cells (Chen et al., 2010). Most preparations of this viral strain have different HA mutations, and importantly, many of these adaptive mutations affect antigenicity. We have overcome this problem by genetically engineering a single HA mutation (D225G) that allows the A/California/7/2009 virus to grow in eggs without further adaptive mutations (Linderman et al., 2014). The D225G HA mutation is antigenically neutral so these viral preparations that have this mutation can be used for accurate antigenic analyses. To illustrate the advantages of this approach, we grew three viral stocks that possessed the wild-type A/California/7/2009 HA and three viral stocks that possessed A/California/7/2009 HA that had the D225G HA mutation (Figure 5). Standard Sanger sequencing revealed that stocks of the “wild-type” A/California/7/2009 had different HA mutations, whereas the stocks engineered to have the D225G HA mutation did not have any additional detectable mutations.

## Materials and equipment

### Biosafety

- Class II Biological Safety Cabinets
- Approved BSL-2 or enhanced BSL-2 laboratory facilities for most mammalian and some avian IAVs. Some highly pathogenic viruses require higher biosafety levels. Identify the proper biosafety level based on the IAV subtypes and ensure that all experiments conform to relevant institutional and governmental biosafety regulations.
- Personal protective equipment (PPE)
- Biohazard trash bags
- Biohazard sharps containers
- Paper towels
- Autoclave
- Ethanol, 70% (vol/vol)

### Reagents

#### Virus culture in eggs

- Embryonated, pathogen-free chicken eggs, 9–11 day old
- Egg sealant (paraffin wax or a melted candle)
- Phosphate buffered saline (PBS) pH7.4 (Life technologies, cat. no. 10010-023)
- Trypticase soy agar (TSA) plate with 5% sheep blood (Thermo Scientific, cat. no. R01200)

#### Cell culture

- MDCK cells (ATCC, cat. no. CCL 334)
- A549 cells (ATCC, cat. no. CCL 185)
- Mycoplasma removal agent (MP BioMedical, cat. no. 093050044 − 5 ml)
- DMEM (Life Tech, cat.no.11995073)
- Trypsin-EDTA, 0.25% (wt/vol) (Gibco, cat. no. 25300054)
- Fetal Bovine Serum (FBS), heat-inactivated (Thermo Scientific, cat. no. 10082-147); 50 ml aliquots should be stored at −20°C and thawed before use.
- Gentamicin Reagent Solution (Gibco, cat. no. 15750-060)
- Sodium pyruvate solution (Invitrogen, cat. no. 11360070)
- L-Glutamine (Invitrogen, cat.no. 25030-081)
- Penicillin/Streptomycin (Invitrogen, cat. no. 15140122)

#### Virus culture in cells

- Bovine albumin 35% solution (BSA) (Sigma, cat. no. A7979)
- TPCK–trypsin (chromatographically purified, lyophilized powder trypsin treated with L-(tosylamido-2-phenyl) ethyl chloromethyl ketone (TPCK)(Worthington Biochemical, cat. no. LS004454TRTPCK)

#### Hemagglutination assay

- Chicken, turkey, or guinea pig red blood cells 5% (vol/vol) (Lampire, cat. no. 7241409, 7249409, 7243109).
- PBS solution-10X ultra-pure (Denville, cat. no. CP4390-48)

#### RNA extraction and PCR for viral genome detection

- TRIzol (Invitrogen; cat. no. 15596018)
- Chloroform (Sigma, cat. no. C2432)
- Ethanol (Decon, cat. no. 64-17-5)
- Isopropanol (Sigma, cat. no. I9516)
- Methanol (Fisher Scientific, cat. no. A412-4) (Chemicals listed above should only be opened in a fume hood or similar safety/exhaust system)
- SuperScript® III One-Step RT-PCR System with Platinum® Taq DNA Polymerase (Invitrogen, cat. no. 12574-026)
- Nuclease-free dH_2_O (Invitrogen, cat. no. 10977-023)
- Primer sequences for one-step PCR for virus detection:
- MB Tuni-12, ACGCGTGATCAGCAAAAGCAGG;
- MB Tuni-13, ACGCGTGATCAGTAGAAACAAGG (optio-nal: use primers specific for each IAV genomic segment)

#### Real-time RT-PCR

- High-Capacity RNA-to-cDNA™ kit (Thermo Scientific, cat. no. 4387406)
- SYBR Green Master Mix (Life Technologies, cat. no. 4368708)
- Primers:
  FLU NP forward, CAGCCTAATCAGACCAAATG;
  FLU NP reverse, TACCTGCTTCTCAGTTCAAG;
  hβ*-actin* forward, AGAGCTACGAGCTGCCTGAC;
  hβ*-actin* reverse, CGTGGATGCCACAGGACT;
  h*GAPDH* forward, GCAAATTCCATGGCACCGT;
  h*GAPDH* reverse, TCGCCCCACTTGATTTTGG;
  h*IFN-b* forward, GTCAGAGTGGAAATCCTAAG;
  h*IFN-b* reverse, ACAGCATCTGCTGGTTGAAG;
  h*IL-29* forward, CGCCTTGGAAGAGTCACTCA;
  h*IL-29* reverse, GAAGCCTCAGGTCCCAATTC;
  h*ISG56* forward, GGATTCTGTACAATACACTAGAAACCA;
  h*ISG56* reverse, CTTTTGGTTACTTTTCCCCTATCC

#### Agarose gel electrophoresis

- Thermo Scientific™ GeneRuler™ 100 bp Plus DNA Ladder 100 to 3000 bp (Fisher, cat. no. SM0322)
- 6X DNA loading dye (Fisher Scientific, cat. no. R0611)
- UltraPure agarose (Invitrogen, cat. no.16500-500)
- dH_2_O, autoclaved and stored at room temperature
- Tris-Acetate-EDTA (TAE), 10X (Invitrogen, cat. no. 15558026)

### Equipment and consumables

#### Common equipment

- Micropipettes (1–1000 μl capacity)
- Multichannel pipettes (8 and 12 channels)
- Pipette controller
- Vortex mixer
- Water bath
- Refrigerated microcentrifuge (for 1.5-ml and 2-ml microtubes)(Eppendorf, cat. no. 5415R or equivalent)
- Refrigerated benchtop centrifuge (for 15-ml and 50-ml conical tubes) (Eppendorf, cat. no. 5810R or equivalent)
- Freezers and refrigerators: −80°C, −20°C, and 4°C
- Ice machine and ice buckets

#### Common consumables

- Sterile glass or plastic bottles (500 ml)
- Sterile polypropylene conical 15 and 50 ml tubes (BD Falcon, cat. no. 352070 or equivalent)
- Sterile screw-cap microtubes, 2 ml (Sarstedt, cat. no. 15071353)
- Snap-cap microtubes, 1.5 ml, autoclaved (Eppendorf, cat. no. 022363204)
- Sterile, aerosol-resistant micropipette tips (1–1000 μl capacity)
- Cotton-plugged, sterile serological pipettes (1–25 ml capacity)
- 0.2 μm and bottle-top microfilters

#### Virus culture in eggs

- Egg trays
- Egg candling light (OvaView or equivalent)
- Egg incubator (Brinsea, OVA-easy 190)
- Moil point chisel
- Syringes (1 ml) with 26G 5/8” needle
- Cotton swabs
- Egg cracker or spoon
- Sterile forceps
- Sterile needle (of 1½)
- Sterile tongue depressor

#### Virus culture in cells

- Tissue culture incubator (37°C, 5–7% CO_2_)
- Straight-neck polystyrene tissue culture flasks with vented caps, 25 and 75 cm^2^
- Polystyrene tissue culture plates with lids, 6, 12, and 96 well plate
- Cell counter
- Cell culture microscope

#### Hemagglutination assay

- Microtiter plates, U-bottom, 96 well (Thermo Scientific, cat. no. 2205)
- Reservoirs (Denville, cat. no. P8826-S)
- Sterile pipette tips of various sizes

#### Real-time RT-PCR

- 384 well plate (Greiner Bio-One, VWR cat. no. 82051-470)
- PCR strip tubes, 0.2 ml (GeneMate, Bioexpress cat. no. T-3035-2)
- ViiA™ 7 Real-Time PCR System with 384-Well Block (Applied Biosystems, cat. no. 4453536)
- Real-time RT-PCR analysis software (ViiA™ 7 Software)

#### Agarose gel electrophoresis

- Microwave
- Glass Erlenmeyer flask, 500 ml
- Agarose gel casting tray
- Electrophoresis chamber
- Power supply
- UV light box
- Imaging system for capturing images of agarose gels

### Reagent setup

- **Embryonated chicken eggs (9–11 day old):** Before inoculation use an egg candle to check that the shell is intact, the egg is fertilized, and the embryo is viable. Mark with a pencil the edge of the air sac.
- **Tissue culture media (TCM):** Sterile DMEM 500 ml, Sodium pyruvate 5 ml (1 mM), L-Glutamine 5.5 ml (2 mM), heat inactivated FBS 50 ml, Gentamicin 500 μl (50ng/ml), filter through a 0.2 μm filter.
- **Infection media (IM):** Sterile DMEM 500 ml, Penicillin/Streptomycin 5 ml, BSA 35% 5 ml, Sodium bicarbonate 5% 12 ml, filter through a 0.2 μm filter.
- **Plaque assay media:** For a total volume of 15 ml necessary for one 6-well plate: DMEM 6.5 ml, DEAE-Dextran 1% 0.3 ml, MgSO_4_ 1.5% 0.3 ml, TPCK-trypsin 0.0075 mg, NaHCO_3_ 7.5% 0.3 ml, Gentamicin 15 μl, Agarose 1.2% 7.7 ml.
- **TPCK-trypsin stock solution (2.5 mg/ml in DMEM):** Dissolve the TPCK-trypsin in DMEM to a concentration of 2.5 mg/ml. Aliquot in 50–200 μl aliquots and keep at −20°C. Before use, thaw the aliquot on ice. Discard after use. The working concentration should always be tested after preparation of a new stock.
- **RBCs, 0.5% (vol/vol), for hemagglutination assay:** The type of RBC used depends on the virus subtype. For example, recent H3N2 clinical isolates do not bind to chicken or turkey RBCs but do still retain some binding to guinea pig RBCs (Klimov et al., 2012). RBCs should be washed with cold PBS before use following procedure below:
  - Transfer 5 ml of blood to a 50 ml tube for centrifugation.
  - Add 45 ml PBS and mix gently.
  - Set the centrifuge acceleration and deceleration rates to 5.
  - Centrifuge 5 min at 1200 rpm in an Eppendorf 5810R refrigerated centrifuge or equivalent.
  - Repeat two or three times until the supernatant is clear.
  - Dilute to 0.5% RBCs in PBS.
- **TAE, 1X for agarose gel electrophoresis:** Mix 10X TAE with dH_2_O at a 1:10 (vol/vol) ratio to the desired volume. 1X TAE can be stored at room temperature for up to 1 year.
- **Agarose gel, 1.5% (wt/vol):** Mix 1.5 g of agarose with 10 ml of 10 × TAE and dH_2_O to 100 ml in a 500-ml glass Erlenmeyer flask. Microwave on high for 1–2 min or until the agarose is completely dissolved. Add and mix in 10 μl Ethidium Bromide solution (EB) per 100 ml gel. Cool the agarose solution slightly, pour it into an agarose gel cast at room temperature and allow the gel to set.

### Equipment setup

- **Class II biological safety cabinet (BSC):** The BSC should be airflow–certified and fully decontaminated with 70% ethanol before use.
- **Tissue culture incubators:** Set incubators to 37°C with an atmosphere of 7% (or 5%) CO_2_ for cell and virus cultures. Supply a tray of water to provide humidity.
- **Egg incubator:** Follow the user's instructions and set the temperature to 37°C and humidity to 45%.
- **Centrifuges:** Cool all centrifuges to 4°C before use.

## Detailed protocol of the procedures

### MDCK cell culture

- Thaw frozen cells in a 37°C water bath.
- After thawing, transfer to a 15 ml conical tube containing 9 ml of TCM.
- Centrifuge 5 min at 1000 rpm in an Eppendorf 5810R refrigerated centrifuge or equivalent to remove the DMSO.
- Resuspend the cell pellet in 1 ml of TCM. (This is assuming a 1 × 10^6^ cell inoculum. Adjust volume to achieve a density of 1 × 10^6^ cells/ml if necessary).
- Transfer to 1 well of a 6-well tissue culture plate. Add 10 μl of Mycoplasma removal agent/1 × 10^6^ cell/ml.
- Upon confluency, release the cells by adding 0.5 ml of 0.25 % trypsin-EDTA followed by a 3 min incubation at 37°C in a tissue culture incubator. Suspend the cells with a pipette and add 1 ml of TCM prior to transfer to a T25 tissue culture flask for expansion. Add another 5 ml of TCM.
- When the cells are confluent 2–3 days later, wash the flask twice with sterile PBS and repeat the trypsinization procedure.
- Transfer cells to a T75 tissue culture flask and add 10 ml TCM. The cells will be ready to be used 2 days later.

### Virus isolation

- One day before virus isolation, seed 5 × 10^5^ MDCK cells / well in TCM in a 6-well plate.
- To ensure proper virus isolation, it is best that plaques are picked from plates containing fewer than 50 plaques. If initial viral titer is unknown, is best to prepare 10-fold serial dilution of the virus in IM from 10^−1^ to 10^−8^ for infection.
- Aspirate the media from the plates containing the cells.
- Wash the cells twice with 1 ml PBS or serum free media.
- Add 400 μl of virus (or virus dilutions) to each well.
- Let the virus adsorb to the cells for 1 h at 37°C in a tissue culture incubator !Caution rock the plates every 15 min to keep wells from drying out.
- During virus adsorption, prepare the plaque assay media. Keep the media at 45–46°C to prevent agarose solidification !Caution hot plaque assay media will damage the MDCK cells.
- After adsorption, aspirate the supernatants using a 1000 μl pipette. If using virus dilutions, start aspirating from most diluted well. Be mindful of potential cross-contamination.
- Wash the cells gently with 1 ml PBS.
- Overlay the cells with 2 ml of plaque assay medium. Add the media carefully. Be mindful of not detaching the cells. Allow the overlay to solidify before placing the plates in the incubator.
- Incubate the plates at 37°C in a tissue culture incubator for 72 h.
- Pick isolated plaques using a 1 ml sterile micropipette tip to pinch through the overlay media.
- Place each plug containing a virus plaque (and agar) in a separate microcentrifuge tube containing 1 ml 0.1% gelatin in PBS. This will be your purified virus stock.
- If the plaqued virus will be expanded on the same day, store the virus at 4°C and continue with the seed virus preparation procedure described below. Otherwise, the plaqued virus stock should be aliquoted in 200 μl aliquots and quick-frozen in a dry ice/ethanol bath and stored at −80°C.

### Seed virus preparation

- One day before virus isolation, seed 2 × 10^5^ MDCK cells in 1 ml TCM per well in 12-well plates.
- Before seeding the virus, aspirate the media from the 12-well plates.
- Wash the cells twice with 1 ml PBS.
- Add 200 μl of purified virus stock to each well.
- Incubate the plates at 37°C in a tissue culture incubator for 1 h. Rock the plates every 15 min to keep wells from drying out.
- Add 1 ml of IM containing 2 μg/ml TPCK-trypsin per well (make sure to titrate each stock of the TPCK-trypsin to avoid disruption of the monolayer, adjust the concentration accordingly) and incubate at 37°C in a tissue culture incubator for 24 h.
- Supernatants should be collected and centrifuged for 5 min at 4°C at 2000 rpm in an Eppendorf 5415R refrigerated centrifuge or equivalent to remove debris.
- Aliquot in 200 μl aliquots and quick-freeze in a dry ice/ethanol bath. Store at −80°C.

### Seed virus characterization and quality control:

- **Virus Titration by TCID50 (Infectivity Titer):**
  - The day before titration prepare 96 well plates with MDCK cells. Seed the plate with 2 × 10^4^ cells / well in TCM (100 μl/well of a solution of 2 × 10^6^ cells / 10 ml TCM). One plate will fit four samples in triplicates. Prepare as many plates as needed.
  - The day of the titration the cells should be 80–90% confluent.
  - In a separate 96 well plate, prepare 1/10 serial dilutions of the virus in triplicates in IM. Add 180 μl of IM to all wells in the plate. Add 20 μl of virus to each well on the first row. Prepare triplicates for each sample. Leave three rows for media alone, and include a positive control with a virus of known titer. Use a multichannel pipette to mix the virus with the media in the first row and transfer 20 μl to the next row. Change tips, mix, and transfer 20 μl to the next row, continue until the last row. Make sure to mix the virus well and change the tips for every dilution. Keep the plate on ice or at 4°C.
  - Discard the media of the plate containing the cells and wash the monolayer twice with 100 μl of PBS. Wash gently to avoid disrupting the monolayer.
  - Using a 12-channel multichannel pipette transfer 25 μl / well of the virus dilutions to the cells and incubate for 1 h at 37°C in a tissue culture incubator. !Caution start transferring virus from the bottom of the plate (higher dilution) to avoid carrying over virus from lower to higher dilutions.
  - Add 175 μl of IM containing 2 μg/ml TPCK-trypsin per well (make sure to titrate each stock of the TPCK-trypsin to avoid disruption of the monolayer, adjust the concentration accordingly) and incubate for 72 h. Be careful not to contaminate the media with virus from the plate.
  - Calculate the virus titer by determining the end point dilution that test positive for hemagglutination of RBCs. To do this, add 25 μl of PBS per well to a 96 well U-bottom plates. Prepare the same number of plates than the number of infected plates. Transfer 25 μl of the cell supernatants to the hemagglutination plate using a multichannel pipette. Transfer starting from the highest dilution. Avoid carry over contamination. Add 50 μl of 0.5% RBCs to each well, mix and incubate for 30 min at 4°C or RT.
  - Score the titer by determining the last dilution with positive hemagglutination. Score the number of positive wells for that last dilution (number of positives out of three). From this score calculate the TCID_50_ / 25 μl using the following formula, where “x” corresponds to the dilution:
    “+++” 10^*X*^ TCID_50_ = 10^*X*+0.7^/25 μl
    “++−” 10^*X*^ TCID_50_ = 10^*X*+0.4^/25 μl
    “+−−” 10^*X*^ TCID_50_ = 10^*X*−0.1^/25 μl
    To obtain the TCID_50_ / ml multiply the calculated titer × 40.
- **Virus Titration by plaque forming units (PFU) (Infectivity Titer):**
  - One day before infection, seed 5 × 10^5^ MDCK cells per well in 6-well plates in TCM. One 6 well plate will be used per each sample. Prepare as many plates as needed.
  - Before infection, prepare 10-fold serial dilutions of the virus in IM.
  - Aspirate the media from the 6-well plates.
  - Wash twice with 1 ml of sterile PBS.
  - Add 400 μl of virus dilution to each well.
  - Adsorb the virus to the cells for 1 h at 37°C in a tissue culture incubator !Caution rock the plates every 15 min to keep wells from drying out.
  - Prepare plaque assay media during the adsorption. Keep the media at 45–46°C to prevent agarose solidification !Caution hot plaque assay media will damage the cells.
  - After the 1 h adsorption, aspirate the supernatant (from most diluted well to more concentrated well). Be mindful not to cross-contaminate dilutions and/or viruses when aspirating.
  - Wash each well with 2 ml of sterile PBS.
  - Add 2 ml of plaque assay media, wait to allow the overlay to solidify before placing in incubator.
  - Incubate the plates at 37°C in a tissue culture incubator for 72 h.
  - Add stationary liquid (acetic acid:methanol:water = 1:4:5) 2 ml per well; incubate the plates at 37°C in a tissue culture incubator for about 1 h.
  - Use a folded syringe needle to remove the agarose overlay.
  - Add crystal violet (1% w/v) 1 ml per well and incubate for 2 min.
  - Wash by submersion in a water bath until all excess crystal violet is gone.
  - Count plaques in the dilution producing around 50 visible plaques and calculate PFU using the following formula in where N is the number of plaques, X is the dilution counted, and V is the virus volume used per well:
  $$\begin{array}{l} PFU∕ml=\frac{N\;\times \;10^{x}}{V} \end{array}$$
- **Hemagglutination Assay (Total titer):**
  - Using a multichannel pipette add 25 μl of PBS to each well of a 96-well U-bottom plate. Prepare as many plates as needed.
  - Add 25 μl of the virus stock /well into the first column of the plate. Each virus stock should be tested in triplicate.
  - Perform a two-fold dilution series across the plate by transferring 25 μl between wells, disposing of the final 25 μl from the last well.
  - Using a multichannel pipette add 50 μl of 0.5% chicken RBCs over all wells, mix and incubate for 30 min at 4°C or RT.
  - Observe endpoint and record the titer per 25 μl of the virus. Positive samples will agglutinate the RBCs. In negative samples RBCs will settle at the bottom of the U-bottom plate forming a tight pellet.
- **Infectivity / Total Particle Ratio Calculation**
  The ratio between Infectivity titer (I) and Total titer (T) equals to TCID_50_ per 25 μl (or PFU) / HA per 25 μl.
- **Testing for the presence of DVGs by PCR:**
  - Extract the RNA from infected cells using TRIzol or other RNA extraction technique.
  - Use 1 μg of RNA from cells infected with seed virus.
  - Prepare Master mix for one step-RT-PCR

**Table:** 

**Reagent**	**Volume μl**
2X Reaction Mix (Invitrogen #12574-018)	12.5
MB Tuni 12 (or specific F primer)	0.5
MB Tuni 13 (or specific R primer)	0.5
SS III RT/Platinum Taq mix	1.0
RNA	1 μg
dH_2_O	to 25 μl total
  - Run PCR program in a thermocycler:

**Table:** 

**Step**	**Temperature (°C)**	**Time**	
1	42	60 min	
2	94	2 min	
3	94	30 s	
4	45	30 s	
5	68	3 min	Go to step 3 (5 cycles)
6	94	30 s	
7	57	30 s	
8	68	40 s	Go to step 6 (35 cycles).
9	10	Forever
  - Add 4 μl of 6X-loading buffer to each sample.
  - Load the samples into a 1.5% agarose gel.
  - If seed virus shows no evidence of DVGs, proceed with virus expansion.
- **Determining if viral stock has prominent mutations**.
  - Extract RNA from viral stock using Qiagen Viral RNA Kit or by using other RNA extraction technique.
  - Create cDNA and PCR amplify gene of interest. HA should always be sequenced and other viral segments can also be sequenced depending on intended use of virus.
  - Gel purify PCR band and sequence product via standard Sanger Sequencing. Illumina sequencing can be completed to identify minor sequence variants.

### Virus propagation

- **Virus amplification in embryonated chicken eggs**
  - Incubate the eggs at 37°C in an egg incubator immediately upon arrival.
  - Work at room temperature one tray of eggs at a time. Return to 37°C as soon as finished. The embryos need to be kept warm to survive.
  - Heat wax or candle until melted.
  - Wipe clean the eggs with an isopropyl alcohol pad. Do not “bath” the eggs in ethanol as the embryo may die due to ethanol toxicity. Work in an aseptic manner.
  - Use a lamp to candle the eggs and mark with a pencil the limit between the air sac and the allantoic cavity.
  - Use a moil point chisel to carefully break the eggshell just enough for a needle to penetrate. Break the shell approximately 1 mm over the limit of the air sac and the allantoic fluid.
  - Inoculate the eggs though the hole with 30,000 TCID_50_ of the virus diluted in 100 μl of PBS. Make sure that the needle is pointing straight down as you want to pass through the membrane and inoculate the virus in the allantoic fluid. Use a 26G 5/8″ needle.
  - Seal the hole with the wax or the melted candle using a cotton swab. Put the eggs back at 37°C in an egg incubator.
  - Wait exactly 40 h. Longer times will produce variable virus stocks due to the generation of defective viruses.
  - Transfer the eggs to 4°C for 3–4 h to kill the embryo.
  - Work in aseptic conditions. Crack the shell open at the air sac using an egg cracker or a spoon. Remove the loosen shell.
  - Using a sterile needle (16G 1½) carefully remove the membrane to expose the allantoic fluid.
  - Using a sterile tongue depressor carefully move the embryo to the side while aspirating the allantoic fluid with a sterile 10 ml pipette. Place the allantoic fluid in a sterile 15 ml conical tube on ice. Allantoic fluid is clear and should not contain yolk (yellowish color). If the yolk sac is disrupted the allantoic fluid should be discarded. A red color indicates RBC contamination and a cloudy appearance indicates bacterial contamination. Change tongue depressor and pipette for each egg.
  - Spin down the allantoic fluid at 4°C 5 min at 2000 rpm in an Eppendorf 5810R refrigerated centrifuge or equivalent to remove debris. A sample of the allantoic fluid should be inoculated in a TSA plate to test for bacterial contamination.
  - Pull the allantoic fluid, mix, and aliquot. Typically, 5–10 mL of allantoic fluid can be isolated from each egg.
  - Aliquots should be snapped frozen in a dry ice/ethanol bath and kept at −80°C.
- **Virus amplification in mammalian cell lines**
  - One day before virus isolation, seed 5 × 10^5^ MDCK cells / well in TCM in a 6-well plate.
  - On the day of inoculation, remove the TCM and wash the cells twice with PBS.
  - Add 400 μl DVGs free virus with IM to achieve a MOI = 0.1.
  - Incubate the plates at 37°C in a tissue culture incubator for 1 h. Shake the plates every 15 min to keep wells from drying out.
  - Add 2 ml of IM containing 2 μg/ml TPCK-trypsin per well (make sure to titrate each stock of the TPCK-trypsin to avoid disruption of the monolayer, adjust the concentration accordingly) and incubate at 37°C in a tissue culture incubator for 24 h.
  - Supernatants should be collected and centrifuged for 5 min at 2000 rpm in an Eppendorf 5810R refrigerated centrifuge or equivalent at 4°C to remove debris.
  - Aliquot in 200 μl aliquots and quick-freeze in a dry ice/ethanol bath and stored at −80°C.

## Important! virus characterization and quality control should be performed for working virus stocks as described above for seed stocks.

### DVG cloning and sequencing

- Prepare LB plates with a final concentration of 100 μg/ml ampicillin following standard protocols.
- Use the gel extraction kit for DNA purification.
- Ligate the DNA to the pGEM-T-easy vector as described below at 16°C overnight.

**Table:** 

**Reagent**	**Volume μl**
2X Rapid Ligation Buffer	5
T4 DNA Ligase	1
DNA insert: pGEM®-T Easy Vector	4 (molecular weight 3:1)
dH_2_O	To 10 μl total
- Transformation: thaw DH5α competent cells on ice.
- Gently mix cells with the pipette tip (do NOT pipette up and down) and aliquot 50 μl of cells for each transformation into 15 ml tubes that have been pre-chilled on ice.
- Add 5 μl ligation mix to the cells and mix gently (do NOT pipette up and down).
- Incubate the tubes on ice for 30 min.
- Heat shock at 42°C for exactly 30 s without shaking.
- Place tubes on ice for 2 min.
- Add 950 μl of LB (make sure this does not contain antibiotics).
- Rotate on an orbital shaker at 37°C for 1 h at 200 rpm.
- Add X-Gal to the plates before using (final concentration of 40 μg/ml X-gal and 0.1 mM IPTG per plate).
- Allow plates to dry and avoid light.
- Centrifuge the DH5α cells 90 s at 10,000 rpm in a microcentrifuge.
- Remove most LB leaving 100-200 μl, resuspend the cells and add to the plate.
- Swab the cells on the plate.
- Incubate the plate at 37°C overnight.
- Number each white colony at the bottom of the plate. Touch each colony with a pipette tip and transfer to a labeled PCR tube for a colony screening. Put the plate at 4°C and perform PCR as described below.

**Table:** 

**Reagent**	**Volume μl**
PCR buffer	2
MB Tuni 12 (or specific F primer)	0.8
MB Tuni 13(or specific R primer)	0.8
SS III RT/Platinum Taq mix	0.1
MgSO_4_	0.6
dNTP	0.4
dH_2_O	15.3 to 20 μl total
- Run PCR program in a thermocycler:

**Table:** 

**Step**	**Temperature (°C)**	**Time**	
1	94	2 min	
2	94	30 s	
3	57	30 s	
4	72	1 min	Go to step 2 (33 cycles)
5	72	10 min	
6	4	Forever
- Run the PCR products in an agarose gel and identify positive colonies.
- Pick positive colonies from the plate with a pipette tip and place them in 2 ml LB with ampicillin.
- Shake overnight on an orbital shaker at 37°C, 200 rpm.
- Extract plasmid using the Promega PureYield Miniprep kit or equivalent.
- Sequence and analyze using the viral genome as reference.

### Immunostimulatory activity

- **RNA extraction**
  Extract total RNA from cell lines using TRIzol or equivalent reagent following manufacturer's specifications.
- **RT-qPCR**
  - Reverse transcribe 1 μg of RNA using the high capacity RNA to cDNA kit from Applied Biosystem or similar system.
  - Dilute the cDNA 1:40 and amplify with specific primers in the presence of SYBR green.
  - Perform qPCR reactions in triplicate using specific primers and the Power SYBR® Green PCR Master Mixture in a Viia7 Applied Biosystem Lightcycler, or similar.
  - Normalize base on levels of β-actin and GAPDH.

## Applications

The procedures described in this protocol avoid the accumulation of the DVGs and adaptive HA mutations during IAV production. Although this is not a clinical protocol, the procedures described are applicable to the propagation and characterization of viruses isolated from clinical samples, as well as for the preparation of virus stocks to be used in the annual preparation of the influenza vaccine. It needs to be kept in mind that some countries have strict regulations in the cell types allowed for vaccine preparation. DVGs and mutations can severely affect the virus replication rate and its virulence. This protocol will help reduce the amount of DVGs that impact the antiviral immune response and may solve problems with virus propagation, as some viral stocks fail to grow to high titers because of their high content of DVGs. Lastly, this protocol may help minimize vaccine mismatch because of the acquisition of adaptive mutations during vaccine preparation. It is important to note that the principles discussed here also apply to the propagation of other RNA viruses although specific materials may vary.

## Anticipated results

**Figure 6:**
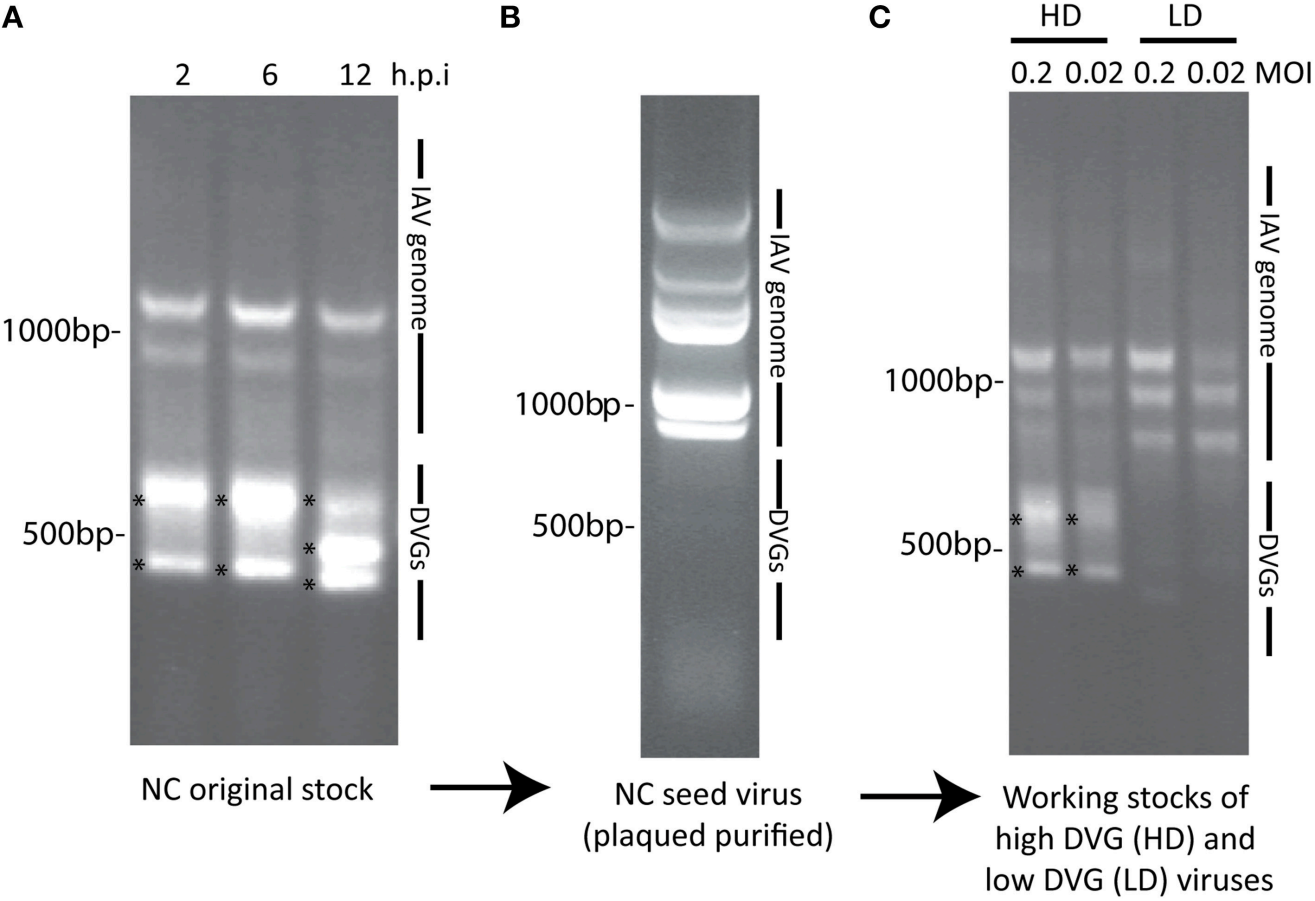
**Example of the generation of a IAV virus stock free of DVGs. (A)** Viral PCR products amplified from A549 cells infected with a MOI 0.1 of a parental stock of A/New Caledonia /03/2005 at different times post infection. **(B)** Viral PCR products amplified from MDCK cells infected for 48 h with A/New Caledonia /03/2005 seed virus prepared after plaque purification at 10^−5^ dilution (around 300 TCID_50_ per well in 6-well plate) from the original stock. **(C)** Viral PCR products amplified from A549 cells infected with different MOIs of working stocks of A/New Caledonia /03/2005 containing a high load of DVGs (HD) or depleted of DVGs (LD) following the proposed protocol. Position of base pair size reference markers is indicated in each gel. Asterisks indicate DVGs confirmed by sequencing.

In previous studies we used IAV strain A/Puerto Rico/8/1934 (PR8) grown following this method to produce stocks with a high or low content of DVGs. Mice infected with IAV PR8 LD showed increased mortality compared to mice infected with IAV PR8 HD and the lungs of mice infected with PR8 HD had higher *Ifnb* mRNA expression indicating that the content of DVGs in IAV stocks affects virulence (Tapia et al., 2013). Here we use A/New Caledonia /03/2005 as an example and show that this procedure is effective in generating virus stocks with minimal DVG contamination. Among all the isolates tested A/New Caledonia /03/2005 generates the highest amount of DVGs and at the fastest rate. As shown in Figure 6, the proposed procedure reduces the number of DVGs in the virus stock to undetectable levels. In data not shown, we have tested various other H1N1 isolates and the mouse adapted X31 (H3N2). In all cases this protocol results in negligible amounts of detectable DVGs at early times post-infection compared to virus expanded in conditions promoting DVG accumulation as illustrated in Figures 1 and 2. As shown in Table 1 the acceptable I/T ratio for different isolates varies largely. It is suggested to establish the acceptable I/T ratio for each isolate by performing the quality control procedures described in this protocol, establish a criterion for acceptable I/T for each isolate, and report this criterion in all related publications. We anticipate that influenza A and B stocks of diverse subtypes will show similar results, albeit the kinetics for reappearance of DVGs may differ.

## Limitations

RNA viruses are replicated by an error prone polymerase that introduces mutations at a rate of 10^−6^ to 10^−4^ substitutions per nucleotide per cell infection (Sanjuan et al., 2010; Regoes et al., 2013). Despite initiating the virus propagation procedure from a single virus plaque, it is important to keep in mind that mutations altering virus fitness may still arise and DVGs may accumulate during the time course of infection. The main goal of this protocol is to establish consistency in the viral stocks used for experimentation and vaccine generation. In our experience, virus amplification following strict conditions consistently results in undetectable levels of DVGs in the viral stocks and after 4–6 h of infection in cell culture. Some viral isolates may have a higher propensity to generate DVGs than others, and this should be taken into consideration. Growing the virus at a lower temperature (35°C) should be tried in this situation. Lower MOIs of infection and shorter propagation times can also be tested, with the caveat of obtaining virus stocks with reduced titer. If DVGs cannot be eliminated following these procedures, the content of DVGs in the virus stock should be reported in all related publications. In addition, because of the low MOI required for the preparation of seed virus without DVGs, the seed virus stock may not reach high titers. Multiple rounds of propagation may be needed to achieve higher titers. The content of DVGs should be checked in each round of propagation to avoid contamination.

## Author contributions

JX performed experiments and wrote the manuscript, BC performed experiments, SH and CL wrote the article and conceived the experiments.

## Funding

Research reported in this publication was supported by the NIAID of the National Institutes of Health under award numbers R01AI083284 and R21AI109472 to CBL, 5T32AI055400 to BC, and 1R01AI113047 and 1R01AI108686 to SH. This research was partially supported by the China Scholarship Council (NO. 201306350082 to JX).

### Conflict of interest statement

The authors declare that the research was conducted in the absence of any commercial or financial relationships that could be construed as a potential conflict of interest.
